# Editorial: The circadian circus – how our clocks keep us ticking

**DOI:** 10.3389/fnins.2022.973727

**Published:** 2022-08-30

**Authors:** Alun Thomas Lloyd Hughes, Hrayr P. Attarian, Jun Hirayama

**Affiliations:** ^1^School of Biological and Environmental Sciences, Liverpool John Moores University, Liverpool, United Kingdom; ^2^Center for Sleep Disorders, Department of Neurology, Northwestern University Feinberg School of Medicine, Chicago, IL, United States; ^3^Department of Clinical Engineering, Faculty of Health Sciences, Komatsu University, Komatsu, Japan; ^4^Division of Health Sciences, Graduate School of Sustainable Systems Science, Komatsu University, Komatsu, Japan

**Keywords:** circadian clock, sleep, metabolism, calcium signal, light, growth hormone - insulin-like growth factor 1 axis, intrinsically photosensitive retinal ganglion cells

Circadian clocks are present in a wide range of organisms, from bacteria to humans, and regulate the organism's basic physiological functions, including sleep, metabolism, and immune activity (Levi and Schibler, 2007; Roenneberg and Merrow, 2016; Eelderink-Chen et al., 2021). Circadian clocks generate self-sustained daily (circadian) rhythms in various biochemical, physiological, and behavioral processes, which run autonomously under constant environmental conditions (Dunlap, 1999; Takahashi, 2017). To ensure that an organism's physiological functions adapt to the rhythm of its environment, circadian clocks are entrained to the external day-night cycles by taking temporal cues from environmental stimuli, such as the daily light-dark variations (Dibner et al., 2010). Indeed, light is the principal cue for the circadian clocks' adaptation (Devlin and Kay, 2001). Disruption of physiological processes is a result of disruption of the clock at the cellular level, which is manifested as inappropriately timed sleep and wakefulness (Baron and Reid, 2014), and has adverse metabolic consequences (Scheer et al., 2009).

In most organisms, circadian clocks are implemented at the molecular level, comprising interconnected transcription–translation feedback loops called cellular clocks (Dunlap, 1999; Takahashi, 2017). Cellular clocks are endogenous cell-autonomous systems capable of maintaining their rhythm in the absence of external time cues. Both vertebrates and invertebrates have cellular clocks throughout their bodies (Schibler and Sassone-Corsi, 2002). In mammals, the circadian system is composed of both central and peripheral clocks (Takahashi, 2017). The anatomical locus of the dominant mammalian central clock is the suprachiasmatic nucleus (SCN), a part of the anterior hypothalamus. The SCN cellular clock integrates light signals received from the retina, then transmits this information to entrain peripheral and other central cellular clocks via both neural and humoral routes (Schibler and Sassone-Corsi, 2002; Okamura, 2004). According to the above schema, circadian clocks can be divided into three conceptual components at the molecular level: (i) pacemakers, generating and sustaining circadian rhythms, and adjusting phase to match the environment; (ii) the input pathways, through which external time cues reach the pacemakers; and (iii) the output pathways, through which circadian clocks affect physiology (Koronowski and Sassone-Corsi, 2021). In this Research Topic, we focus on the molecular and physiological mechanisms underlying the regulation of these three components. In particular, we aim to better understand the manner in which regulatory defects lead to physiological and behavioral deficits that in turn lead to disease.

The molecular mechanisms of the input pathway that mediate light signaling to the SCN are of central importance. In mammals, in particular, there are intrinsically photosensitive retinal ganglion cells (ipRGCs) that use the photopigment melanopsin to detect light and transmit this information through their afferents to the central cellular clock in SCN (Aranda and Schmidt, 2021). Stinchcombe et al. develop mathematical models of the electrical activity of ipRGCs to show how ipRGC-to-SCN network connectivity is tuned to allow for accurate light-dependent regulation of the circadian clock in mice. Circadian clocks are affected by an enormous variety of stimuli known to trigger intracellular signaling pathways, including pathways mediated protein kinase C, glucocorticoid, Wnt, tumor growth factor-b/activin, mitogen-activated protein kinases, calcium, and growth hormone (GH) (Schibler and Sassone-Corsi, 2002; Hirota and Fukada, 2004; Uchida et al., 2010). Conversely, several studies have demonstrated at the molecular level how these intracellular signaling mediators contribute to circadian clock regulation. In particular, a large number of studies have indicated the interaction between cellular clocks and signaling pathways mediated by calcium or GH that may result in the functional coupling of the circadian clocks and physiological processes. Cavieres-Lepe et al. review the reciprocal relationship between the calcium signaling pathway and circadian clocks in *Drosophila* and mice. In particular, they describe key roles that the calcium signaling pathway plays in the regulation of the light input pathway, central circadian pacemakers, and output pathways of the circadian clocks. Furthermore, they discuss the discovery of the converse, i.e., the regulation of the calcium signaling pathway by circadian clocks, and this could improve drug efficacy and postoperative clinical outcomes. GH is a key promoter of somatic growth and partakes in the regulation of substrate metabolism (Kraemer et al., 2020). GH exerts its metabolic action in multiple tissues, including the liver, muscle, fat, and pancreas, either directly or indirectly through insulin-like growth factor 1 (IGF-1) (Kraemer et al., 2020). Wang et al. review the current clinical and animal evidence on the interaction of circadian clocks with the GH—IGF-1 axis and discuss its association with current public health problems, especially metabolic disorders caused by circadian clock dysregulation.

In our society of a round-the-clock lifestyle, many people engage in jobs on the nightshift and develop sleep rhythm disorders. Exposure to light at inappropriate times during the sleep wake cycle, as it tends to happen when people work night shift, suppresses melatonin. Disruption in melatonin rhythms results in dysfunction of physiological processes such as the GH-IGF-1 axis in animals (Kraemer et al., 2020). The ensuing weight gain, metabolic disorders, and related cardiovascular and cerebrovascular diseases have become a major public health problem (Evans and Davidson, 2013). Chronic mistimed lighting events, such as night-time light exposure, are becoming increasingly common. However, relatively little is known about the impact of long-term exposure to subtle disruptive conditions such as dim light at night (DLAN), a representative feature of modern living conditions for most of the population. Delorme et al. evaluate the effects of long-term exposure of mice to abnormal lighting conditions, including DLAN, revealing that DLAN leads to altered circadian behavior and motor function. Moreover, the same authors provide evidence that these phenotypes are mediated by changes in neuronal coordination that does not involve the central clock in the SCN.

The circadian clock regulates the timing of sleep in humans (Lee et al., 2021). A major and growing social problem in our time is insufficient or delayed sleep that can lead to fatigue, poor performance, and health problems. Procrastination is a voluntary, irrational delay in performing an intended activity despite awareness of the negative effects of postponing it (Steel, 2007). When procrastination is stable over time, it should be considered a personality trait. Herzog-Krzywoszanska et al. reveal the significance of bedtime procrastination in the relationship between personality characteristics and daytime fatigue. These insights suggest new directions for possible therapeutic interventions by targeting specific personality traits of the individuals who do not go to bed on time and consequently experience daytime fatigue.

The organization of modern industrial society has created a situation in which humans are forced to adapt their wakeful hours to the demands created by social incentives. This often leads to disruptive lifestyles, such as working shifts and habitual night-time eating, disrupting the circadian clock and inducing a variety of diseases. Thus, it is critical that we gain a better understanding of the association of diseases with the dysregulation of circadian clocks by environmental factors in modern society, and develop lifestyle intervention strategies to maintain proper circadian clock regulation.

## Author contributions

JH: writing—original draft. AH and HA: writing—review and editing. All authors contributed to the article and approved the submitted version.

## Funding

This Research Topic and editorial was supported in part by grants to JH [The Japan Society for the Promotion of Science (JSPS) Grant-in-Aid for Scientific Research (B) No. 20H04565 and the Kobayashi foundation] and to AH [The Physiological Society (Research Grant)].

## Conflict of interest

The authors declare that the research was conducted in the absence of any commercial or financial relationships that could be construed as a potential conflict of interest.

## Publisher's note

All claims expressed in this article are solely those of the authors and do not necessarily represent those of their affiliated organizations, or those of the publisher, the editors and the reviewers. Any product that may be evaluated in this article, or claim that may be made by its manufacturer, is not guaranteed or endorsed by the publisher.
